# Validation of the Brazilian Portuguese version of the Quick Inventory of Depressive Symptomatology and Self-Report (QIDS-SR_16_) for the Brazilian population

**DOI:** 10.47626/2237-6089-2020-0378

**Published:** 2023-05-19

**Authors:** Lucas Bandinelli, Julia Luiza Schäfer, Bruno Kluwe-Schiavon, Rodrigo Grassi-Oliveira

**Affiliations:** 1 Programa de Pós-Graduação em Psicologia Pontifícia Universidade Católica do Rio Grande do Sul Porto Alegre RS Brazil Programa de Pós-Graduação em Psicologia, Pontifícia Universidade Católica do Rio Grande do Sul (PUCRS), Porto Alegre, RS, Brazil.; 2 Developmental Cognitive Neuroscience Lab Instituto do Cérebro PUCRS Porto Alegre RS Brazil Developmental Cognitive Neuroscience Lab, Instituto do Cérebro, PUCRS, Porto Alegre, RS, Brazil.; 3 Programa de Pós-Graduação em Psiquiatria e Ciências do Comportamento Universidade Federal do Rio Grande do Sul Porto Alegre RS Brazil Programa de Pós-Graduação em Psiquiatria e Ciências do Comportamento, Universidade Federal do Rio Grande do Sul, Porto Alegre, RS, Brazil.; 4 CICPSI Faculdade de Psicologia Universidade de Lisboa Lisboa Portugal CICPSI, Faculdade de Psicologia, Universidade de Lisboa, Lisboa, Portugal.; 5 Translational Neuropsychiatry Unit Department of Clinical Medicine Aarhus University Aarhus Denmark Translational Neuropsychiatry Unit, Department of Clinical Medicine, Aarhus University, Aarhus, Denmark.

**Keywords:** Depressive symptoms, symptom evaluation, self-assessment, clinical psychology

## Abstract

**Objectives:**

To evaluate the psychometric properties of the Quick Inventory of Depressive Symptomatology (QID-SR16), a self-report instrument based on the Diagnostic and Statistical Manual of Mental Disorders, 4th edition (DSM-IV) criteria that assesses the severity of depression symptoms, in the Brazilian population.

**Methods:**

Participants were 4,400 Brazilians over the age of 15 years recruited for an online survey assessing depressive symptoms during the early phase of the coronavirus disease 2019 (COVID-19) pandemic in Brazil. The internal consistency, construct validity, and convergent and discriminant validity of the QIDS-SR_16_ were evaluated.

**Results:**

The model tested was considered an adequate fit to the data (comparative fit index [CFI] = 0.947, Tucker-Lewis index [TLI] = 0.927, and root-mean-square error of approximation [RMSEA] = 0.051) and its internal consistency was good, with a Cronbach’s alpha of 0.71 and an average item correlation of 0.23. The correlations between the total QIDS-SR_16_ score and the total scores of the Patient Health Questionnaire (PHQ-9) instruments (r = 0.67, p < 0.001), the Posttraumatic Symptoms Checklist (PCL-5) (r = 0.61, p < 0.001), and the Patient-Reported Outcomes Measurement Information System (PROMIS) (r = 0.60, p < 0.001) indicate good concurrent and convergent validity.

**Conclusion:**

The QIDS-SR_16_ has robust psychometric properties in terms of its internal consistency, construct validity, and convergent and discriminant validity. The Portuguese version of the QIDS-SR_16_ is an adequate instrument for assessment of depressive symptoms in the context of an online survey.

## Introduction

In 2020, the World Health Organization (WHO) estimated that depression was the second leading cause of disability in the world and it is responsible for significant impairment of people’s functionality and quality of life.^1^ It is a serious disorder, with incidence that increases year on year, and is associated with considerable morbidity and increased mortality in the general population.^2,3^ There are 322 million people living with depression in the world and it is more common among women (5.1%) than among men (3.6%)WHO,^4^ In Brazil, there are few population-based studies to precisely estimate the prevalence of depression. However, a national study with approximately 3,000 participants found that the prevalence of depressive symptoms was 28.3% of participants, with 15.3% of them occurring in depressive episodes considered severe.^5^

The severity of depressive symptoms is an important factor to consider in initial assessments, requiring use of brief and effective instruments that provide the health professional with this data, since treatment guidelines can be established on the basis of this information, leading to greater effectiveness.^6^ Several of these assessments are made in primary care settings, which is often where the population first accesses and comes into contact with health professionals, and an estimated 19.5% of depression cases are diagnosed at this level of care.^7^ A multicenter study carried out in four Brazilian cities (Rio de Janeiro, São Paulo, Fortaleza, and Porto Alegre) found that, respectively, 25, 25.3, 31, and 21.4% of patients seen at basic health units had a diagnosis of depression or significant symptoms of the disorder.^8^ Despite this apparently high prevalence, it should be emphasized that many false positives can occur because of the difficulty of making these diagnoses for general practitioners with little training in mental health.^9^ It can therefore be concluded that access to simple and adequate instruments is important to help health professionals who do not have specific training in mental health to conduct more accurate assessments of depressive symptoms.

Within this perspective, many scales and inventories have been widely used, such as the Beck Depression Inventory (BDI-II), the Center for Epidemiological Studies Depression (CES-D) scale,^10^ the Patient Health Questionnaire (PHQ-9),^11^ the Depression, Anxiety, and Stress Scales (DASS),^12^ the Hospital Anxiety and Depression Scale (HADS),^13^ and the Hamilton Depression Rating Scale (HAM-D6),^14^ but they usually require trained professionals and are time consuming. Thus, the Quick Inventory of Depressive Symptomatology and Self-Report (QIDS-SR_16_), derived from the Inventory of Depressive Symptomatology-Self-Report (IDS-SR_30_),^15^ emerges as an option to enable initial assessment of depressive symptoms to be conducted quickly and efficiently, since it focuses only on the nine criteria necessary for a diagnosis of major depressive disorder contained in the Diagnostic and Statistical Manual of Mental Disorders, 4th edition (DSM-IV), and is easy for the general population to understand.

In addition, the QIDS-SR_**16**_ seems to offer some advantages in relation to the other scales. When compared with the CES-D and the DASS, it has a closer relationship with the DSM criteria for depression, greater sensitivity to change, and better assessment of the risk of suicide.^16^ A study comparing the sensitivity and specificity of scales for depression in primary care observed that the QIDS-SR_**16**_ had greater specificity for assessment of major depression and minor depression than the PHQ-9 (84.7% vs. 72.2%).^17^ The HADS may be an appropriate scale for assessment of depressive symptoms, but it ends up excluding the disorder’ somatic symptoms, suppressing a dimension that can be important in this initial assessment and which is covered by QIDS-SR_**16**_.^18^ The BDI-II is one of the main instruments for evaluation of depression and has good correlations with the QIDS-SR_16_.^19^ However, in Brazil it is too expensive for use as a tool for initial screening in primary health care settings. In addition, it must be considered that, according to our legislation (Resolução CFP 009/2018), the BDI-II can only be used by experienced psychology professionals, who are not always available at primary care units.

The QIDS-SR_16_ scoring system converts the responses to the scale’s 16 items into nine domains based on the DSM-IV diagnostic criteria for depression: 1) sad mood, 2) poor concentration, 3) self-criticism, 4) suicidal ideation, 5) anhedonia, 6) energy/fatigue, 7) sleep disturbance, 8) decrease/increase in appetite/weight, and 9) psychomotor agitation/retardation.^19^ Each item can be scored on a response scale from 0 to 3, on which respondents choose the score that best describes them in the last 7 days. The QIDS-SR_16_ total score ranges from 0 to 27, with higher values indicating greater severity of depressive symptoms.

Thus, QIDS-SR_16_ constitutes an important screening tool to identify primary care patients who may meet the diagnostic criteria for major depressive disorder^20^ that is easier for health professionals to use,^21^ since it requires minimal training for application because it is a self-administrated instrument.^19^ However, considering that no validation studies exist for the Brazilian population, this study aims to evaluate the psychometric properties of construct validity, internal consistency, and concurrent and convergent validity of the Brazilian Portuguese version of the QIDS-SR_16_ scale.

## Methods

### Data collection

This is a cross-sectional and observational study. The data used were collected from April 18 to May 11, 2020, in an online survey using the Qualtrics platform, the main objective of which was to collect information about the impact of coronavirus disease 2019 (COVID-19) on stress, trauma, and risk perception in the Brazilian population. Any Brazilian, over the age of 15, residing in Brazil or abroad, could respond to the survey by accessing a link made available on various social networks on the internet. The data presented in the present study are therefore derived from this primary study and are part of a more comprehensive research project. For this study in particular, participants were selected aged 18 to 65 years who had completed all of the scales used for the validation process, which were presented to them in the following order: PHQ-9, QIDS-SR_16_, PROMIS, and PCL-5. Only the participants who scored above the cutoff point of the PHQ-9 scale (≥ 13), indicating the presence of depressive symptoms, were included in the validation analysis. All participants were recruited through electronic media (for example, social networks, websites, blogs, etc.) using the snowball sampling method, in which the researcher invites participants to share the survey with their contacts. The sample size was calculated using the public domain program OpenEpi (www.openepi.com), adopting a 95% confidence level, a 1% margin of error, and a random sample. A set of criteria was applied to maximize data reliability. Initially, participants who took less than 5 minutes to complete the survey were excluded. Then, with regard to socioeconomic variables, participants who provided invalid information about age, zip code, and the last four digits of their mobile numbers were excluded (only the last four digits were requested to avoid identifying participants). Subsequently, since in this study we were not interested in investigating changes that occurred in participants over time, possible repeated measures were excluded by checking for both repeated zip codes and the last four cellphone digits.

### Instruments

#### Quick Inventory of Depressive Symptomatology-Self-Report (QIDS-SR16)

The QIDS-SR_16_ is a brief scale for assessing depressive symptoms based on the DSM-IV diagnostic criteria that is derived from the IDS-SR_30_ scale, originally developed in English. The IDS-SR_30_ was adapted to Brazilian Portuguese, exhibiting good psychometric properties.^22^ The QIDS-SR_16_ has been translated into 31 languages,^23^ including Brazilian Portuguese.^24^ It has 16 items in total, grouped into nine domains (sad mood, poor concentration, self-criticism, suicidal ideation, anhedonia, energy/fatigue, sleep disturbance, decrease/increase in appetite/weight, and psychomotor agitation/retardation). The scores for three domains (sleep disturbance, decrease/increase in appetite/weight and psychomotor agitation/retardation) are based upon the maximum score (most pathological) of two or more questions. Each of the remaining domains is rated by a single item. All domains are scored from 0 to 3, with higher scores indicating greater psychopathology. Total QIDS scores range from 0 to 27, with scores of 5 or lower indicative of no depression, scores from 6 to 10 indicating mild depression, scores from 11 to 15 indicating moderate depression, scores from 16 to 20 reflecting severe depression, and total scores greater than 21 indicating very severe depression.

#### Patient Health Questionnaire (PHQ-9)

The PHQ-9 scale is an instrument for assessment of depression in primary care, and is available in Portuguese. It consists of nine questions, which correspond to the nine diagnostic criteria for depression. Each item can receive up to four responses (0-3 points), indicating the frequency of the presence of symptoms in the last 2 weeks. At the end of these nine questions, respondents are asked about the impact these symptoms have had on their functionality. The total score ranges from 0 to 27 and represents the sum of the responses of the nine items.

#### Patient-Reported Outcomes Measurement Information System (PROMIS)

The PROMIS questionnaire^25^ enables assessment of aspects of anxiety involving the dimensions fear (fear, panic), anxious anguish (worry, dread), hyperexcitation (tension, nervousness, restlessness), and somatic symptoms associated with arousal (fast heart, dizziness). The abbreviated eight-item form was used, in which it is necessary to indicate the frequency of symptoms related to anxiety on a Likert scale from 1 (never) to 5 (always).

#### Posttraumatic Symptoms Checklist (PCL-5)

The PCL-5^26^ is a self-report scale comprising 20 items with a Likert response scale ranging from 0 = nothing to 4 = a lot. The instrument aims to measure the severity of symptoms and provide a diagnosis of post-traumatic stress disorder. Severity scores can be calculated for each symptom within each of the clusters: (B) intrusions, (C) avoidance, (D) negative changes in cognition and mood, and (E) increased excitability; or for any disorder by the sum of the items. Individuals who score more than 44 points are considered to have high levels of symptoms of post-traumatic stress and individuals who score 44 or less have low levels of symptoms.

## Ethical aspects

Research participants were invited to complete the online questionnaire anonymously and voluntarily and had to indicate their consent by reading and accepting the Free and Informed Consent Form. Participants were not paid for their participation. This research project was approved by the National Research Ethics Commission (Conselho Nacional de Saúde [CONEP], 30502620.4.0000.0008).

## Data analysis

First, analyses of central tendency and variability were conducted to describe the sample and the variables of interest and to evaluate the distribution of the data. As a means of testing construct validity as measured by the QIDS-SR_16_, a confirmatory factor analysis was performed, testing a single-factor model of depressive symptoms. The adequacy of the model was assessed, considering as adequate values above 0.90 for the comparative fit index (CFI) and the Tucker-Lewis index (TLI) and a value below 0.06 for the root-mean-square error of approximation (RMSEA).^27,28^ The instrument’s internal consistency was assessed using Cronbach’s alpha analyses, considering values between 0.70 and 0.79 as acceptable, 0.80 and 0.89 as good, and results above 0.90 as excellent,^29^ and by analyzing the average correlation between items. Ideally, the average correlation between items should be between 0.20 and 0.40, suggesting reasonable homogeneity and significant single variance between items.^30^

Finally, concurrent and convergent validities were investigated using Spearman’s correlation analyses, considering correlation coefficients less than 0.30 as low, between 0.30 and 0.50 as moderate, and above 0.50 as high.^31^ Concurrent validity is determined by comparing the scores on an instrument of interest (in this case, the QIDS-SR_16_) with the scores on a reference instrument measuring the same construct (in this case, the PHQ-9). Convergent validity is assessed by comparing scores on the instrument of interest with scores on another instrument measuring a related but different construct (in this case the PCL-5 and the PROMIS).^32^ Correlation coefficients above 0.40 were considered to be adequate indicators of validity. All analyses were performed using *R* software.

## Results

A total of 8,825 people answered the online survey and 49% (n = 4,400) of these scored above the PHQ-9 cutoff point, constituting the sample used for validation of the instrument. The average age was 33.0 (standard deviation [SD] = 10.93) years, 83% (n = 3,644) of respondents were female, 49% (n = 2,170) were single, and 50% (n = 2,213) had postgraduate university qualifications. Details of the sociodemographic data for the sample and the mean scores for each of the instruments are presented in Table 1.

**Table 1:** Sample description (n = 4,400)

	n (%)				
Sex: female	3,644 (83)				
Marital status					
Single	2,170 (49)				
Married	1,209 (28)				
Divorced	306 (7)				
Widowed	30 (0.7)				
Stable relationship	685 (16)				
Education					
Elementary school	11 (0.2)				
High school	538 (12)				
Graduate	1,638 (37)				
Postgraduate	2,212 (50)				
	
	**Mean (SD)**	**Min**	**Max**	**Skew**	**Kurtosis**

Age	33 (10.93)	18	65	0.67	-0.26
Total PHQ-9 score	14.45 (4.69)	7	27	0.56	-0.54
Total QIDS score	9.45 (4.12)	0	24	0.38	-0.16
Total PROMIS score	21.1 (5.74)	7	35	-0.11	-0.57
Total PCL-5 score	43.48 (13.75)	20	100	0.82	0.34
PCL Re-experiencing	9.91 (4.08)	5	25	1	0.52
PCL Avoidance	4.16 (1.94)	2	10	0.83	0.02
PCL Cognition/mood changes	15.55 (5.54)	7	35	0.77	0.09
PCL Arousal	13.86 (4.48)	6	30	0.67	0.07

PCL = Posttraumatic Symptoms Checklist; PHQ-9 = Patient Health Questionnaire; PROMIS = Patient-Reported Outcomes Measurement Information System; QIDS = Quick Inventory of Depressive Symptomatology; SD = standard deviation.

### Construct validity

**Figure 1:**
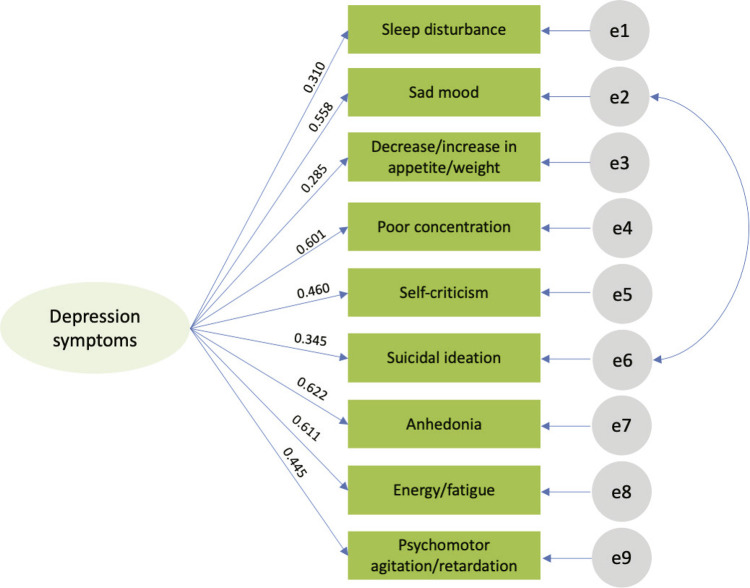
Single-factor model of depressive symptoms composed of the nine symptoms measured by QIDS-SR16

A single-factor model of depressive symptoms composed of all nine symptoms measured by QIDS-SR_16_ (Figure 1) was tested with confirmatory factor analysis using full maximum information likelihood estimation. The model tested was considered adequate to the data according to the indexes χ^2^ (26, n = 4,400) = 325.376, p < 0.001, CFI = 0.947, TLI = 0.927, and RMSEA = 0.051. More information about the item factor loadings is presented in Table 2.

**Table 2:** QIDS-SR16 factor loadings (n = 4,400)

QIDS-SR_16_ dimensions	β	SE	p-value	CI
Sleep disturbance	0.310	0.016	< 0.001	0.278-0.341
Sad mood	0.558	0.013	< 0.001	0.532-0.584
Decrease/increase in appetite/weight	0.285	0.016	< 0.001	0.253-0.317
Poor concentration	0.601	0.013	< 0.001	0.576-0.626
Self-criticism	0.460	0.015	< 0.001	0.431-0.489
Suicidal ideation	0.345	0.016	< 0.001	0.313-0.376
Anhedonia	0.622	0.012	< 0.001	0.598-0.647
Energy/fatigue	0.611	0.013	< 0.001	0.586-0.635
Psychomotor agitation/retardation	0.445	0.015	< 0.001	0.416-0.474

CI = confidence interval; QIDS-SR_16_ = Quick Inventory of Depressive Symptomatology and Self-Report; SE = standard error.

### Internal consistency

The QIDS-SR_16_ demonstrated good internal consistency with a Cronbach’s alpha of 0.71 and an average correlation coefficient between items of 0.23.

### Concurrent and convergent validity

The correlations between the total QIDS-SR_16_ score and the total scores of the PHQ-9 (r = 0.67, p < 0.001), PCL-5 (r = 0.61, p < 0.001), and PROMIS (r = 0.60, p < 0.001) instruments indicate good concurrent and convergent validity. Analyzing the correlations between the total QIDS-SR_16_ score and the posttraumatic stress disorder dimensions assessed by the PCL-5, it can be observed that the correlation coefficient for the dimension changes in cognition and mood (r = 0.63, p < 0.001) is higher than the coefficients for re-experiencing (r = 0.41, p < 0.001), avoidance (r = 0.31, p < 0.001), and arousal (r = 0.57, p < 0.001).

## Discussion

The original study that developed and validated the QIDS-SR_16_ demonstrated its usefulness for evaluation of depressive symptoms and proved its psychometric validity.^15^ This instrument has been tested in a variety of settings for assessment of depressive symptoms, such as for assessment of young adult students at universities,^33^ in veteran military personnel with comorbid post-traumatic stress disorder,^34^ and in patients with bipolar mood disorder.^35^ Its usefulness for screening for depressive symptoms in primary health care is emphasized^13,17,20,36^ and it can be an important instrument within this context.

In this study, we sought to validate the Brazilian translation of the QIDS-SR_16_ and evaluate its psychometric properties in order to make this instrument available to health professionals who work in primary care in the country and also in other mental health care settings in general. When comparing our results for the scale’s internal consistency process with the results of the original English version, we observe that our results reveal a lower Cronbach’s alpha (0.86 in the original and 0.71 in our study), but one that is still within the acceptable range and is not so far from the results of validation studies of the Chinese (0.73),^37^ German (0.77),^38^ and Korean (0.73) versions.^39^

The differences in these values in relation to the original version and to the other versions cited can be explained by the sample size used in each of the translation validation processes. In our study, we had a total of 4,400 respondents, which is a much larger number than in the validation process for the original version (n = 596)^19^ and also than in the other validation studies. We also emphasize that our study analyzed a symptomatic population in the context of the pandemic, since people were directed to fill out the QIDS-SR_16_ after an initial screening using the PHQ-9 scale, which differs from the other validation studies of the scale.^19,40,41^ We could infer that the fact that the collection took place online and not face-to-face as in the other translation validation processes could interfere with our results, although several studies have shown that there are no significant differences in the quality of the data collected when administration via these two modalities is compared. Moreover, online research may even be more advantageous, because in theory it allows for more sincere responses from participants.^42,43^

All of the correlations between the total QIDS-SR_16_ score and the instruments assessing depressive symptoms (PHQ-9), post-traumatic symptoms (PCL), and anxiety (PROMIS) are considered high, supporting its concurrent and convergent validity. These results are related to findings in the literature in which QIDS-SR_16_ has already shown high correlations with the PHQ-9 (r = 0.81^39^) and with symptoms of anxiety (r = 0.603).^38^ Notwithstanding, when examining the relationship between the total QIDS-SR_16_ score and the PCL dimensions, we noted that the greatest correlations were with the group of symptoms related to mood and to changes in cognition. Together, these results indicate that QIDS-SR_16_ should also be a good tool for assessment of depressive symptoms in screening processes.

Despite its satisfactory results, our study has some limitations that merit mention. The first concerns the fact that our sample comes from a survey in which the original main objective was to evaluate the traumatic and stress reactions of the Brazilian population during the COVID-19 pandemic and this context may have some influence on the rate of positive responses. However, it is believed that an efficient scale should have similar results in different contexts, with the possibility of varying only the intensity of the symptoms and not their constructs. Also regarding the sample, the participants had a high educational level (50% had postgraduate degrees), which does not necessarily represent the Brazilian population in general. Another issue is that 83% of the participants were female, which makes it more difficult to generalize these data for both sexes. However, it is observed that there is an important sex-difference in rates of depression diagnosis, with a higher prevalence in women than in men.^44^

Considering the high incidence of diagnoses of depressive conditions in Brazil in primary care outpatient clinics^8^ and the adequacy of the psychometric properties of the Brazilian Portuguese translation of QIDS-SR_16_, it is concluded that this instrument may be able to assist health teams in assessment of and screening for depressive symptoms, without requiring minimal preparation for this, since it is not always possible to count on the presence of a mental health professional in this health care sector.
